# Norovirus in benign convulsions with mild gastroenteritis

**DOI:** 10.1186/s13052-016-0303-2

**Published:** 2016-11-03

**Authors:** Gun-Ha Kim, Jung Hye Byeon, Deog-Yong Lee, Hyun Ju Jeong, Baik-Lin Eun

**Affiliations:** 1 Department of Pediatrics, Korea University College of Medicine, Seoul, South Korea; 2 Division of Enteric Diseases, Center for Infectious Disease, National Institute of Health, Korea Center for Disease Control & Prevention (CDC-Korea), Chungcheongbuk-do, South Korea; 3 Department of Pediatrics, Korea University Guro Hospital, 148, Gurodong-ro, Guro-gu, Seoul 08308 South Korea

**Keywords:** Seizures, Gastroenteritis, Norovirus, Pediatric

## Abstract

**Background:**

Benign convulsions with gastroenteritis (CwG) are defined as afebrile convulsions accompanying symptoms of gastroenteritis without evidence of laboratory derangement. Although the main pathogen has been known as rotavirus, since the introduction of rotavirus vaccine, associated viruses with CwG may have changed. Thus, we evaluated the viral association of CwG for patients admitting for recent 2.5 years.

**Methods:**

All patients hospitalized for CwG between November 2012 and May 2015 were included in our study. Stool specimens were tested with reverse transcription polymerase chain reaction for detecting norovirus and astrovirus and with enzyme immunoassay for rotavirus and enteric adenovirus. Clinical data was gathered via chart review.

**Results:**

Fifty patients were included. Except four patients who failed to collect stool samples, 46 patients were tested. Causative diarrheal viruses were detected in 38 patients and they were 29 norovirus, four rotavirus, four adenovirus, and one astrovirus. Norovirus was commonly identified during the months of November and December. No difference of the clinical characteristics and laboratory value was noted according to the number of seizure episodes.

**Conclusions:**

Norovirus is a common pathogen in CwG. Understanding the viral associations can facilitate recognition of CwG.

## Background

Benign convulsions with gastroenteritis (CwG) are defined as afebrile convulsions accompanying symptoms of gastroenteritis without evidence of laboratory derangement and have an excellent prognosis [1, 2]. The main causative pathogen of CwG has been known as rotavirus. Since rotavirus vaccine has been introduced in Korea since 2007 (RotaTeq in 2007 and Rotarix in 2008) and viral association of CwG could have changed. Similar to US [1], a recent nation-wide survey in Korea reported that norovirus was the most prevalent pathogen in acute gastroenteritis, followed by rotavirus [2]. A small case series in Korea also reported that norovirus was more prevalent than rotavirus in CwG [3]. Thus, we evaluated the viral association of CwG for patients admitting for recent 2.5 years.

## Methods

### Study population

All hospitalized patients diagnosed with CwG at the Korea University Guro Hospital during 2.5 years between November 2012 and May 2015 were included.

Referring the published papers [3, 4], CwG was defined as follows: a) seizures accompanying symptoms of gastroenteritis; b) no hypoglycemia or electrolyte imbalance; c) no focal neurologic signs; d) no specific abnormalities on EEG or magnetic resonance imaging; and d) no history of unprovoked seizure. To rule out febrile seizures or epilepsy, patients with (a) fever (>37.5 °C) during the 12 h before and after seizures or (b) recurrent seizures during the following 6 months were excluded. Clinical and laboratory data were gathered via a chart review.

### Virus detection

Stool specimens were routinely screened for the presence of norovirus and astrovirus using reverse transcription polymerase chain reaction, and for the presence of rotavirus and enteric adenovirus using enzyme immunoassays, at the National Institute of Health, Korea Center for Disease Control and Prevention. The specimens were also sent to the microbiology laboratory for bacterial culture of *Salmonella*, *Shigella*, and *Campylobacter* species, to exclude bacterial gastroenteritis.

### Statistical analysis

We used Mann − Whitney *U* test for comparison of continuous variables. *P* < 0.05 was considered statistically significant. Analyses were performed with SPSS (ver. 19.0; SPSS Inc., Chicago, IL, USA).

## Results

### Microorganisms identified in CwG

Total 50 patients were diagnosed with CwG, as shown in Table 1. We failed to collect stool samples of four patients. Consequently, 46 stool specimens were tested for viral study and bacterial culture. Causative diarrheal viruses were detected in 38 (82.61 %) of the fecal specimens. Norovirus was the most prevalent pathogen (29 of 46, 63.04 %), followed by rotavirus (4 of 46, 8.70 %), enteric adenovirus (4 of 46, 8.70 %), and astrovirus (1 of 46, 2.17 %). All bacterial culture results were negative.

**Table 1:** Identification of pathogens in patients with benign convulsions associated with mild gastroenteritis

Number of patients	50
Not tested	4
Tested	46
Norovirus	29 (63.04 %)
Rotavirus	4 (8.70 %)
Enteric adenovirus	4 (8.70 %)
Astrovirus	1 (2.17 %)
No virus	8 (17.39 %)
Bacteria	0

### Seasonal distribution of viral association with CwG

**Fig. 1:**
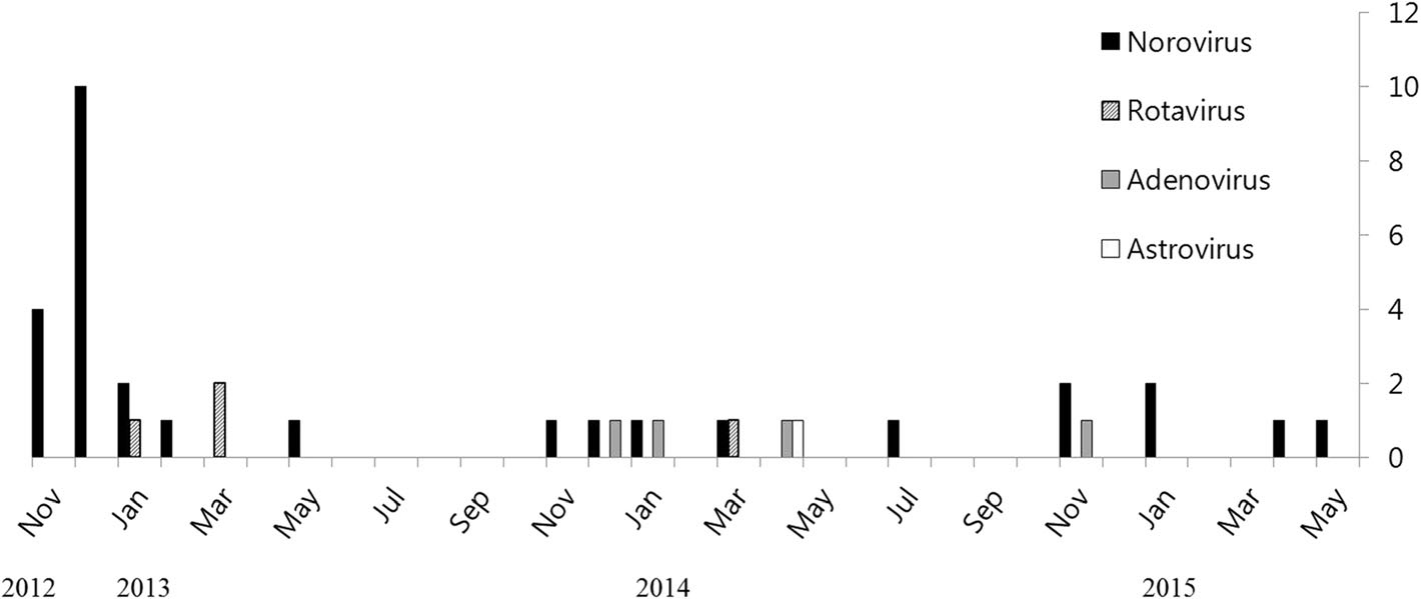
Seasonal distribution of viral association with benign convulsion with gastroenteritis. Norovirus was commonly identified during November and December

As shown in Fig. 1, patients were admitted from November through March of each year. Norovirus was commonly identified during the months of November and December. Seasonal predominance was not evident with other viruses.

### Clinical characteristics of norovirus-associated CwG

**Fig. 2:**
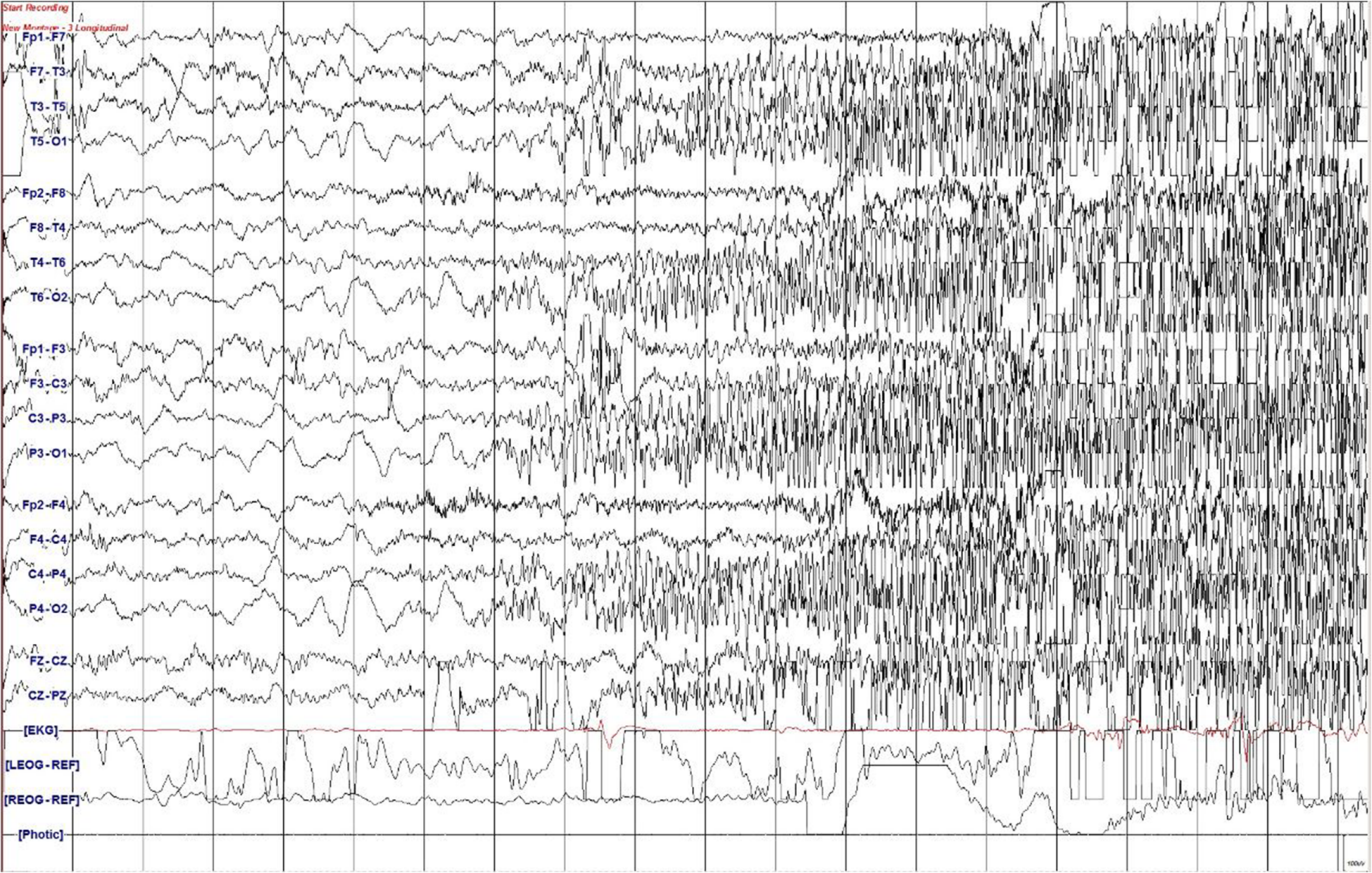
Ictal EEG recording in a patient with benign convulsion with mild gastroenteritis. Bilateral posterior onset, rhythmic, fast activities are rapidly spreading to entire hemispheres

The total number of patients with norovirus-associated CwG was 29; 19 ± 5.39 (mean ± SD) months of age; nine males and 20 females (Table 2). Most of these viruses belonged to genogroup II (28 of 29, 96.55 %). Latency from gastroenteritis to seizure onset was 43.62 ± 18.69 (mean ± SD) hours. Seizures are described as generalized in all patients. Interictal electroencephalogram (EEG) showed transient diffuse (two patients) or focal (two patients) slow waves that were normalized on the following tests. We captured 1 ictal event showing bilateral, posterior onset, rhythmic activities, rapidly spreading to both hemispheres (Fig. 2) and the EEG showed no interictal epileptiform discharges. Duration of seizure episode was 2.41 ± 2.10 (mean ± SD) minutes. The mean number of seizure events was 2.79 ± 2.82 (mean ± SD), while two of the patients had more than ten seizure events within 5 h (one patient had 11 seizures in 5 h, and the other patient had 13 seizures in 4.5 h). The average elapsed time between the first and the last seizure was 3.45 ± 4.87 (mean ± SD) hours. Intravenous lorazepam was introduced in nine patients and the mean hospital stay was 4.07 ± 3.16 (mean ± SD) days.

**Table 2:** Clinical data of patients with Norovirus-positive CwG (*n* = 29)

Genogroup I/II,^n^	1/28
Age (months)	19.40 ± 5.39
Male/female	9/20
Latency to seizure onset (hours)	43.62 ± 18.69
Seizure semiology	
Focal with secondary generalization	0
Apparently generalized	29
Interictal EEG	
Focal or diffuse slow waves	4
Normal	25
Seizure duration (minutes)	2.41 ± 2.10
Number of seizures	2.79 ± 2.82
Time-span of clusters (hours)	3.45 ± 4.87
Antiepileptic use (n)	9
Hospital days	4.07 ± 3.16

Values are the mean ± SD unless otherwise indicated; *n* number of patients

### Comparison of clinical and laboratory parameters according to the number of seizures in norovirus-associated CwG

We divided patients with norovirus-associated CwG into two groups according to the total number of seizures (< 5 times and ≥5 times) (Table 3) and compared the clinical and laboratory variables between the groups. There was no significant difference in age, latency to seizure onset, duration of hospital stay, and laboratory values between the groups of higher and lower number of seizures.

**Table 3:** Comparison of clinical and laboratory parameters according to the number of seizures

	Number of seizures		*P*-value
	<5	≥5	
Number of patients	20 (68.97 %)	9 (31.03 %)	
Age (months)	17.70 ± 4.79	21.33 ± 5.92	0.93
Latency to seizure onset (hours)	45.20 ± 18.13	40.11 ± 20.55	0.63
Hospital days	3.40 ± 1.70	5.56 ± 4.96	0.19
Laboratory value			
Leukocytes (/uL)	7944.50 ± 2631.63	8655.56 ± 3153.61	0.76
CRP (mg/L)	9.18 ± 12.99	18.64 ± 29.51	0.75
pH	7.34 ± .063	7.37 ± .09	0.46
Base deficit	7.67 ± 3.47	5.04 ± 6.14	0.49
Uric acid (mg/dL)	9.27 ± 1.95	8.18 ± 2.04	0.30
BUN (mg/dL)	14.35 ± 4.49	11.40 ± 3.83	0.07
Creatine (mg/dL)	0.26 ± 0.05	0.24 ± 0.03	0.26
Na (mmol/L)	134.95 ± 2.26	134.78 ± 1.40	0.53
K (mmol/L)	4.31 ± 0.46	4.478 ± 0.3232	0.42

Values are the mean ± SD unless otherwise indicated; *CRP* C-reactive protein

## Discussion

In our study, norovirus was the most prevalent virus during the study period, followed by rotavirus, enteric adenovirus and astrovirus. Although detection rate of norovirus was lower in our study than that of reported case series (15 of 18 patients) [5], it was notable that the norovirus was predominantly associated with CwG. Park et al. reported that rotavirus was detected in 8 of 50 CwG patients during 2010 to 2014 [6]. Of 42 rotavirus-negative patients, further viral studies were performed among 21 patients and norovirus was identified in 15 of 21 patients. Although they did not perform routine viral screening for norovirus, a significant number of patients were positive for norovirus.

Increasing rate of rotavirus immunization and norovirus infection could explain our results. The immunization rate of rotavirus in Korea was reported about 30 % in 2009, and reached 50.2 % in 2012 [7, 8]. In addition, a recent nation-wide survey reported that norovirus was the most prevalent pathogen in acute gastroenteritis (9.7 %), followed by rotavirus (5.0 %), and other viruses (< 2 %) [2].

Seasonally, norovirus was more commonly identified during November and December in our study, while not evident with other viruses. According to the previous report [9], incidence of norovirus peaks in November and December, whereas rotavirus is more prevalent from January to May. Latency from the symptom onset of gastroenteritis to seizure, the average elapsed time between the first and the last seizure and the number of seizures in norovirus-associated CwG were similar with those previously reported for rotavirus-associated CwG [9, 10]. Although we could not analyze the difference of the clinical characteristics according to the associated pathogens, other authors reported that the younger age of onset and longer duration of seizures was observed among the CwG patients with norovirus than in the patients with rotavirus. With regard to the frequency of seizure, no difference was noted between norovirus and rotavirus. Carbamazepine treatment shortened the time span of the seizures especially for norovirus associated CwG [9].

Ictal onset has been demonstrated as focal even if convulsions are described mostly as generalized as our study [11, 12]. We also captured a focal-onset seizure rapidly evolving to both entire hemispheres on ictal recording (Fig. 2) while seizure semiology was generalized. Seizures can be variable during episodes even for the same patient [12–14].

Of interest, two extreme cases were noted among the norovirus-associated CwGs in our study; one patient suffered 11 seizures over a period of 5 h and another had 13 seizures over a period of 4.5 h. To compare the difference of clinical pictures according to number of seizures, we divided the norovirus-associated CwG patients into two groups with higher and lower number of seizures but no significant difference was found. We assume that the explosive seizures might be related to a larger viral load or to different viral strains of norovirus. Or it could be a peculiar presentation of norovirus associated CwG. The stool of a patient with an active norovirus infection contained 100 billion virus particles/g of feces [15], which is 10 times greater than that seen in rotavirus infection [16]. To elucidate this assumption, viral load should be measured according to the number of seizures and multicenter studies to compare clinical features between norovirus and rotavirus-associated CwGs are needed.

Intravenous lorazepam appeared to be ineffective in our study. Several clinical trials have also mentioned the lack of efficacy of benzodiazepine in CwG [17, 18]. Other than benzodiazepines, several authors reported beneficial effect of lidocaine and carbamazepine [9, 17, 19]. Still, there is no consensus regarding drug of choice and the need for treatment. Prospective, controlled clinical trials are needed to demonstrate the necessity and efficacy of anticonvulsants.

Although we could not follow up the patients for years, clinical and neuropsychological outcome of CwG is known as excellent. Recent study reported that none of 81 CwG patients developed epilepsy and only mild attention deficit was detected in less than 5 % of patients with mean follow-up duration of 9.8 years [20].

Our study has some limitations. Clinical difference between norovirus- and rotavirus-associated CwG was not studied due to small number of rotavirus cases. In addition, we only performed norovirus genogrouping, not genotyping. Future studies could compare clinical differences according to genotyping in norovirus-associated CwGs.

## Conclusions

Norovirus is a common pathogen in CwG. Understanding the viral associations can facilitate recognition of CwG.

## References

[1] Payne DC, Vinjé J, Szilagyi PG, Edwards KM, Staat MA, Weinberg GA (2013). Norovirus and medically attended gastroenteritis in US children. N Engl J Med.

[2] Hwang BMLD, Chung GT, Yoo CK (2015). Laboratory Surveillance of Viral Acute Gastroenteritis in Korea, 2014.

[3] Verrotti A, Tocco A, Coppola G, Altobelli E, Chiarelli F (2009). Afebrile benign convulsions with mild gastroenteritis: a new entity?. Acta Neurol Scand.

[4] Castellazzi L, Principi N, Agostoni C, Esposito S (2016). Benign convulsions in children with mild gastroenteritis. Eur J Paediatr Neurol.

[5] Chan CM, Chan CW, Ma CK, Chan HB (2011). Norovirus as cause of benign convulsion associated with gastro-enteritis. J Paediatr Child Health.

[6] Park SH, Kim YO, Kim HK, Kim HS, Kim BY, Cheon KR (2015). Incidence of benign convulsions with mild gastroenteritis after introduction of rotavirus vaccine. Brain and Development.

[7] Lee SG, Jeon SY, Kim KY. Korea National Immunization Survey. 2012. Available at http://www.cdc.go.kr/CDC/info/CdcKrInfo0201.jsp?menuIds=HOME001-MNU1155-MNU1083-MNU1375-MNU0025&cid=20768. Accessed 5 Oct 2016.

[8] Choe YJ, Yang JJ, Park SK, Choi EH, Lee HJ (2013). Comparative estimation of coverage between national immunization program vaccines and non-NIP vaccines in Korea. J Korean Med Sci.

[9] Kawano G, Oshige K, Syutou S, Koteda Y, Yokoyama T, Kim B-G (2007). Benign infantile convulsions associated with mild gastroenteritis: a retrospective study of 39 cases including virological tests and efficacy of anticonvulsants. Brain and Development.

[10] Kang B, Kim DH, Hong YJ, Son BK, Kim DW, Kwon YS (2013). Comparison between febrile and afebrile seizures associated with mild rotavirus gastroenteritis. Seizure.

[11] Cusmai R, Jocic-Jakubi B, Cantonetti L, Japaridze N, Vigevano F (2010). Convulsions associated with gastroenteritis in the spectrum of benign focal epilepsies in infancy: 30 cases including four cases with ictal EEG recording. Epileptic Disord.

[12] Verrotti A, Nanni G, Agostinelli S, Parisi P, Capovilla G, Beccaria F (2011). Benign convulsions associated with mild gastroenteritis: a multicenter clinical study. Epilepsy Res.

[13] Imai K, Otani K, Yanagihara K, Li Z, Futagi Y, Ono J (1999). Ictal Video‐EEG Recording of Three Partial Seizures in a Patient with the Benign Infantile Convulsions Associated with Mild Gastroenteritis. Epilepsia.

[14] Maruyama K, Okumura A, Sofue A, Ishihara N, Watanabe K (2007). Ictal EEG in patients with convulsions with mild gastroenteritis. Brain and Development.

[15] Atmar RL, Opekun AR, Gilger MA, Estes MK, Crawford SE, Neill FH (2008). Norwalk virus shedding after experimental human infection. Emerg Infect Dis.

[16] Greenberg HB, Estes MK (2009). Rotaviruses: from pathogenesis to vaccination. Gastroenterology.

[17] Tanabe T, Okumura A, Komatsu M, Kubota T, Nakajima M, Shimakawa S (2011). Clinical trial of minimal treatment for clustering seizures in cases of convulsions with mild gastroenteritis. Brain Dev.

[18] Okumura A, Uemura N, Negoro T, Watanabe K (2004). Efficacy of antiepileptic drugs in patients with benign convulsions with mild gastroenteritis. Brain Dev.

[19] Okumura A, Tanabe T, Kato T, Hayakawa F, Watanabe K (2004). A pilot study on lidocaine tape therapy for convulsions with mild gastroenteritis. Brain and Development.

[20] Verrotti A, Moavero R, Vigevano F, Cantonetti L, Guerra A, Spezia E (2014). Long-term follow-up in children with benign convulsions associated with gastroenteritis. Eur J Paediatr Neurol.

